# Letter to the editor “Protective immune cell phenotypes and breast cancer risk: insights from Mendelian randomization and meta-analysis”

**DOI:** 10.1097/JS9.0000000000003570

**Published:** 2025-10-06

**Authors:** Jun Li, Honggang Shao

**Affiliations:** Department of Urology, Mianyang Central Hospital, School of Medicine, University of Electronic Science and Technology of China, Mianyang, China

*Dear Editor,*

Breast cancer remains the most common malignancy among women worldwide, with its occurrence, progression, and prognosis closely linked to the tumor immune microenvironment. Recently, Xu *et al*^[1]^ conducted a large-scale Mendelian randomization (MR) analysis combined with meta-analysis to systematically evaluate the causal relationships between 731 immune cell phenotypes and breast cancer risk. Their results demonstrated that CD3 expression on CD28^+^ CD4^−^/CD8^−^ T cells and HLA-DR expression on CD33^−^/HLA-DR^+^ cell subsets were significantly inversely associated with breast cancer risk, suggesting a potential protective role for these immune phenotypes.

This study exhibits methodological rigor. First, it integrated two independent large-scale genetic databases – BCAC and FinnGen R10 – encompassing more than 140 000 breast cancer cases, ensuring adequate sample size and robust statistical power. Second, multiple MR approaches, including inverse variance weighting, MR-Egger regression, and the weighted median method, were employed, followed by Bonferroni correction and reverse MR validation. These steps collectively minimized potential confounding and reverse causality, thereby strengthening causal inference. Third, using large-scale population genetic data, this work for the first time identified a significant inverse association between these immune cell phenotypes and breast cancer risk, providing new insights into breast cancer immunology. Finally, the findings carry potential clinical implications, as the identified immune phenotypes may inform risk stratification, immune profiling, and novel therapeutic target development, aligning with the rapid advances in precision oncology and immunotherapy.

The clinical significance of this study is noteworthy. Incorporating immune cell phenotypes into risk assessment models could facilitate early identification and personalized management of high-risk populations. Moreover, therapeutic strategies aimed at enhancing the activity of these immune cell subsets, either alone or in combination with immune checkpoint inhibitors such as PD-1/PD-L1 blockade, may further improve clinical outcomes. In addition, the abundance and functional status of these immune phenotypes could serve as potential biomarkers for predicting treatment response and long-term prognosis, thereby aiding clinical decision-making.

Nevertheless, several aspects warrant further investigation. The study population was primarily of European ancestry, lacking validation across Asian, African, and other ethnic groups, thus limiting the generalizability of the findings. Furthermore, the absence of molecular subtype information (e.g., Luminal, HER2-positive, triple-negative breast cancer) and treatment outcome data precluded analyses stratified by tumor subtype. The study also relied on genetic epidemiological inference and lacked functional validation *in vitro* or *in vivo*, leaving the underlying biological mechanisms unresolved. Additionally, immune cell phenotypes may vary dynamically with disease stage and therapeutic interventions, whereas the cross-sectional nature of the genetic data limits assessment of such temporal dynamics.

Future research should address these limitations in several ways. Large-scale, multicenter prospective studies across diverse populations are needed to validate the robustness and generalizability of the findings. Integration of single-cell transcriptomics, spatial transcriptomics, and animal models will be essential to elucidate the mechanistic roles of these immune phenotypes in the tumor microenvironment. Clinical studies should be designed to assess the therapeutic value and safety of strategies aimed at enhancing the activity of these immune cell subsets in combination with immune checkpoint inhibitors. Finally, incorporating immune phenotype data with molecular subtype, treatment response, and long-term outcome information will help advance precision immunotherapy for breast cancer.

In conclusion, this study, leveraging large-scale genetic evidence, identifies two immune-related phenotypes potentially exerting protective effects against breast cancer. These findings provide novel insights into the immunological mechanisms underlying breast cancer and offer a theoretical basis for developing immunotherapy strategies. Despite limitations in population diversity, functional validation, and clinical stratification, the exploratory and innovative nature of this work is commendable and lays the groundwork for future mechanistic research and clinical translation.

## Data Availability

All data analyzed or generated during this study are included in the article.
